# Reduction of Pressure Pain Sensitivity as Novel Non-pharmacological Therapeutic Approach to Type 2 Diabetes: A Randomized Trial

**DOI:** 10.3389/fnins.2021.613858

**Published:** 2021-03-11

**Authors:** Jens Faber, Ebbe Eldrup, Christian Selmer, Caroline Pichat, Sofie Korsgaard Hecquet, Torquil Watt, Svend Kreiner, Benny Karpatschof, Finn Gyntelberg, Søren Ballegaard, Albert Gjedde

**Affiliations:** ^1^Endocrine Unit, Department of Medicine, Herlev Gentofte University Hospital, Herlev, Denmark; ^2^Faculty of Health and Medical Sciences, University of Copenhagen, Copenhagen, Denmark; ^3^Steno Diabetes Center Copenhagen, Gentofte, Denmark; ^4^Institute of Biostatistics, University of Copenhagen, Copenhagen, Denmark; ^5^Department of Psychology, University of Copenhagen, Copenhagen, Denmark; ^6^The National Research Center for the Working Environment, Copenhagen, Denmark; ^7^Department of Neuroscience, University of Copenhagen, Copenhagen, Denmark; ^8^Translational Neuropsychiatry Unit, Aarhus University, Aarhus, Denmark

**Keywords:** type 2 diabetes, glucose homeostasis, glucose control, HbA1c, autonomic dysfunction, non-pharmacological intervention, pressure pain sensitivity, lateral hypothalamus

## Abstract

**Background:**

Autonomic nervous system dysfunction (ANSD) is known to affect glucose metabolism in the mammalian body. Tradition holds that glucose homeostasis is regulated by the peripheral nervous system, and contemporary therapeutic intervention reflects this convention.

**Objectives:**

The present study tested the role of cerebral regulation of ANSD as consequence of novel understanding of glucose metabolism and treatment target in type 2 diabetes (T2D), suggested by the claim that the pressure pain sensitivity (PPS) of the chest bone periosteum may be a measure of cerebral ANSD.

**Design:**

In a randomized controlled trial of 144 patients with T2D, we tested the claim that 6 months of this treatment would reduce PPS and improve peripheral glucose metabolism.

**Results:**

In the active treatment group, mean glycated hemoglobin A1c (HbA1c) declined from 53.8 to 50.5 mmol/mol (intragroup *p* = 0.001), compared with the change from 53.8 to 53.4 mmol/mol in the control group, with the same level of diabetes treatment but not receiving the active treatment (between group *p* = 0.036). Mean PPS declined from 76.6 to 56.1 units (*p* < 0.001) in the active treatment group and from 77.5 to 72.8 units (*p* = 0.02; between group *p* < 0.001) in the control group. Changes of PPS and HbA1c were correlated (*r* = 0.37; *p* < 0.001).

**Conclusion:**

We conclude that the proposed approach to treatment of T2D is a potential supplement to conventional therapy.

**Clinical Trial Registration:**

www.clinicaltrials.gov (NCT 03576430).

## Introduction

An estimated 400 million people worldwide have type 2 diabetes (T2D), and the number may rise to 600 million in 2045 according to the World Health Organization (WHO), with huge and rising social costs. Present treatments include medication and lifestyle adjustments of diet and physical exercise.

The autonomic nervous system (ANS) regulates functions of the human body by adjusting the balance between two opposing and interacting systems, the sympathetic and parasympathetic nervous systems. ANS dysfunction (ANSD), i.e., sympathetic predominance, is associated with the development of T2D, impaired glucose metabolic regulation, and poor outcome of T2D, including increased comorbidity (e.g., cardiovascular disease, renal insufficiency, and peripheral neuropathy) and increased mortality (Vinik et al., 2013; Lundqvist et al., 2019; Spallone, 2019). Similarly, ANSD is linked to human obesity and excessive body fat, insulin resistance, and sleep apnea, all precursors of T2D (Peterson et al., 1988; Saito et al., 2015; Kakutani-Hatayama et al., 2020).

The conventional concept of glucose homeostasis as regulated entirely outside the brain has been found to be too simple. The region in the brain responsible for ANS control of glucose homeostasis is the hypothalamus, among other sites (Arrigoni et al., 2019; Lundqvist et al., 2019). A recent study of mice by magnetic resonance imaging *in vivo* demonstrated increased metabolic activity of the lateral hypothalamus by infusion of glucose (Mohr et al., 2020). Thus, specific nuclei in the hypothalamus regulate peripheral glucose concentration, either by means of the ability of the nuclei to sense glucose concentrations through the floor of the third ventricle (Zhang and van den Pol, 2016; Arrigoni et al., 2019; Lundqvist et al., 2019) or by means of afferent nerve impulses from peripheral organs sensitive to changes of peripheral glucose concentrations, including the pancreas (Güemes and Georgiou, 2018). The efferent stimulus with effects on beta- and alpha-cells in the pancreas seems a balance between the effects of glucose-excited and glucose-inhibited neurons in the hypothalamus (Wang et al., 2004; Thorens, 2011) and efferently mediated by the sympathetic and parasympathetic nervous systems acting through the ANS (Güemes and Georgiou, 2018; Lundqvist et al., 2019).

Excess sympathetic activity has been proposed as an important contributor to T2D through disturbed glucose metabolism by increased insulin resistance (Lundqvist et al., 2019). Thus, hypothetically, peripheral nerve stimulation modulating the central ANS regulating centers would be a novel therapeutic approach to diabetes management, with special focus on reduction of the sympathetic activity, but no information on the potential efficacy of such treatment has yet been reported (Güemes and Georgiou, 2018). As such, the present study is the first test of this approach in patients with T2D.

Pressure pain sensitivity (PPS) is a measure of the threshold of pain and discomfort sensation at the chest bone, in response to a gradually increased pressure during a period of 3–5 s. The concept of PPS originally arose from the observation that individuals with chronic ischemic heart disease or a previous stroke had increased sensitivity to digital palpation of the chest bone during subsequent periods of increased stress sensation (Ballegaard et al., 1999, 2004; Magnusson et al., 2010). Subsequently, an algometer was developed to quantify the PPS of the chest bone (Ballegaard et al., 2009).

Cross-sectional studies in healthy individuals and in patients with ischemic heart disease show that the elevation of PPS at the chest bone periosteum (but not at the index finger periosteum) is related to measures of persistent stress, including depression, clinical symptoms of stress, and reduced well-being (Ballegaard et al., 2012, 2014, 2015; Bergmann et al., 2013, 2014; Vanìèek et al., 2017). PPS also has been found to be associated with cardiovascular health risk factors that are regulated by the ANS, including heart rate (HR), blood pressure (BP), work of the heart measured as the pressure-rate product (systolic BP multiplied by HR; PRP), body mass index (BMI), visceral fat, and serum levels of cholesterol, triglyceride, and glycated hemoglobin (HbA1c) (Ballegaard et al., 2014). The tilt table test as a measure of autonomic function in people with stable ischemic heart disease (Ballegaard et al., 2016) showed that PPS measured both at rest and during a table tilt test is related to the resting HR, the HR variability (HRV), and the baroreflex response to tilting. Further reduction of PPS by clinical intervention trials was associated with improvement of the physiological and biochemical health factors listed above (Axelsson et al., 2014; Ballegaard et al., 2014, 2016).

We originally proposed intervention by reduction of elevated PPS measures, based on, (1) daily cognitive reflection using home PPS measurement as a behavioral guide to stress reduction, (2) repeated non-painful sensory nerve stimulation on specific areas of the body, and (3) ongoing professional surveillance (Bergmann et al., 2014). The intervention lowered PPS and at the same time limited the important diabetes risk factors of level of stress, ANSD activity, BP level, pulse rate, serum lipid concentrations, and depression, as demonstrated in randomized controlled trials (RCTs) in patients with stable ischemic heart disease and in healthy volunteers (Ballegaard et al., 2012, 2015; Bergmann et al., 2014). In office workers, a reduction of an elevated PPS has been found to be strongly associated with a corresponding reduction of an elevated HbA1c (Ballegaard et al., 2014).

Furthermore, and surprisingly, in contrast to other outcome measures, including depression score, baroreflex sensitivity, and HRV, the PPS measure failed to reflect beta-adrenoceptor blockade that generally is held to inhibit the efferent stress response mediated by the ANS (Ballegaard et al., 2016). The failure is consistent with the hypothesis that the PPS measure reflects sympathetic autonomic function emanating from brain centers placed more centrally than the baroreflex of the medulla oblongata, regulated by mechanisms not involving beta-adrenergic receptors (Meller et al., 2016). Beta-adrenergic receptors are located at many sites of the brain, in addition to the lateral hypothalamus (Rainbow et al., 1984). However, in the lateral hypothalamus, the orexin cells regulate stress responsiveness, metabolic and circadian homeostasis, and pain perception (Arrigoni et al., 2019) but remain unaffected by beta-receptor blockade (Murakami et al., 2015).

First, in the present randomized intervention study, we tested the hypotheses that the proposed intervention reduces both HbA1c and PPS in patients with T2D and that the reduction of PPS is correlated with reduction of HbA1c (i.e., study 1). Second, we assessed the possible association between PPS and sympathetic ANSD in T2D patients and the proposed effect of beta-receptor blockade medication with respect to a possible identification of the location of the PPS control center. Autonomic neuropathy is highly prevalent in T2D as evidenced by changes of resting HR and HRV in response to stand-up, deep breathing, and the Valsalva maneuver (Spallone, 2019). Accordingly, we tested whether the chest bone PPS is linked to the presence of cardiovascular autonomic neuropathy (CAN) in patients with T2D (primary endpoint) and whether this association and the PPS measure are unaffected by beta-receptor blockade (secondary endpoint).

## Materials and Methods

### Effect of Pressure Pain Sensitivity Reduction in Type 2 Diabetes

#### Ethics

The study was approved by the local ethical committee (ID) (identifier: H 17034836), the Danish Data Protection Agency, and was registered on www.clinicaltrials.gov (identifier: NCT 03576430). All participants gave their written informed consent after oral and written information about the study. The study was performed according to the Declaration of Helsinki. The original protocol, the study data for the present paper, and a consort checklist are all available upon request.

#### Study Population

**FIGURE 1:**
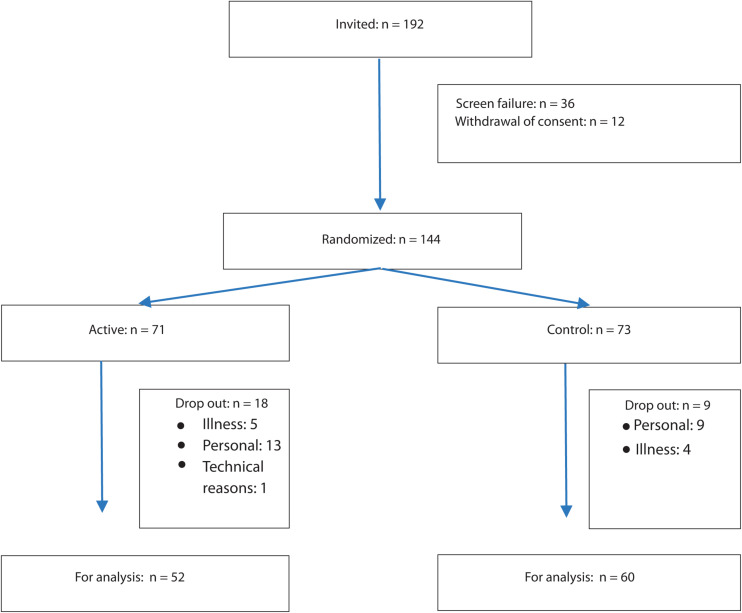
Consort diagram.

We selected patients with mildly to moderately dysregulated T2D, with relatively mild or no complications, i.e., patients typically followed up by their general practitioner rather than hospital clinics. Patients with T2D were recruited from a community-based study (the Herlev-Oesterbro study, Copenhagen, Denmark). The study was carried out from January 2018 until February 2020. During this study, all participants were asked for consent to contact the participant in case of a new scientific study. One hundred ninety-two consecutive participants agreed to be contacted again and were invited to participate in the present study. Of these, 144 persons with T2D fulfilled the criteria (Figure 1) for inclusion and none for exclusion.

#### Inclusion Criteria

(1) A diagnosis of T2D, (2) HbA1c ≤ 75 mmol/mol (≤ 9%), (3) BMI < 40 kg/m^2^, (4) PPS ≥ 60 arbitrary units, (5) age between 18 and 75 years, (6) the ability to manage the Danish language for proper use of instructions, and (7) actively express to accept to conduct minimum 20 min of self-care daily. The cutoff point: PPS ≥ 60 arbitrary units for categorization of a person as having increased PPS and thus signs of ANSD and/or persistent stress was based on several consecutive studies on risk factors for impaired health (Ballegaard et al., 2012, 2015; Bergmann et al., 2014).

#### Exclusion Criteria

(1) Use of insulin as basal-bolus regimen (i.e., the combination of long-term acting insulin as basal insulin and short-term lasting insulin as bolus injections before meals); (2) use of beta blockade medication; (3) previously diagnosed and treated for a psychiatric disorder, except for depression; (4) a chronic competing disorder that statistically is life-shortening (such as advanced cancer); (5) a chronic competing disorder that is not a heart disease and which clearly impairs the person’s quality of life, e.g., chronic obstructive pulmonary disease (COPD), cancer, and chronic pain syndrome; (6) persons who cannot fend for themselves; and (7) one or more of the following complications: diabetic retinopathy that needs specific treatment, or nephropathy (i.e., plasma creatinine ≥ 200 μmol/L; urinary albumin excretion rate was not used for exclusion, as this parameter is subject to fluctuations over time).

#### Randomization

After the baseline visit, the participants were allocated randomly with a 1:1 ratio to intervention or control, with the latter group receiving conventional treatment. The participants were sequentially stratified into three groups: HbA1c < 53 mmol/mol (< 7%): HbA1c = 53–63 mmol/mol (7–8%); HbA1c = 64–75 mmol/mol (8–9%), in order to obtain a balanced distribution regarding HbA1c in the two groups. The randomization was performed and carried out by an independent research assistant using computerized randomization, the result being unknown to the investigators. The participants received the allocation result by a confidential e-mail. Seventy-one participants were allocated to active intervention and 73 to control.

#### Study Protocol

Before and after 6 months, all participants came to the metabolic ward. Outcome measures were obtained by a study nurse blinded to the randomization result at the Medical Department, Endocrinology Unit, Herlev Gentofte Hospital, Denmark. After 10-min rest in the supine position, the PPS was recorded; and subsequently, resting BP and HR, height, and body weight were measured. Finally, a blood sample was obtained.

#### Outcome Measures

The primary outcome measure was HbA1c. The secondary outcome measures were (i) PPS and (ii) the correlation between changes in PPS and changes in HbA1c. HbA1c was measured for treatment evaluation at baseline and after 6 months of observation. It was also measured after 3 months of observation for the evaluation of diabetes medication only. Blood samples were taken locally at Medical Department, Endocrinology Unit, Research Laboratory 54O4, Herlev Gentofte Hospital, Denmark. Routine analyses as HbA1c were measured immediately.

#### Pressure Pain Sensitivity

The Ull Meter (Ull Care Ltd., Denmark) measures the sensibility in the polymodal sensory nervous system (i.e., the pressure pain threshold) at the most tender point of the sternum between costae 3 and 5, reflecting the area of segmental innervation of the heart (Williams et al., 1995), identified by finger pressure. After 10 min of rest in the supine position, the participant first learns the technique and becomes familiar with the procedure by measurement twice on the control point of the dorsal part of the middle phalanx of the left index finger. Here, the study nurse applies manually a gradually increasing pressure with the instrument until the participant says stop when the threshold of pain/discomfort is reached, in total allowing up to 5-s pressure time. If a withdrawal reflex is observed, typically, the startle reflex from the eyes, this is considered a stop signal as well. The procedure is then repeated at the tenderest place of the sternum, identified by palpation by the observer. The procedure at the sternum is conducted twice, and the PPS measure is calculated as the mean of the two recordings. However, if the two recordings differ by more than 10 PPS arbitrary units, a third measurement is conducted, and the PPS measure is calculated as the mean of the three recordings. The instrument displays a number on a scale from 30 to 100 (indicative of pressure pain thresholds from approximately 400 to 25 kPa, i.e., a factor 16 in difference), where increasing sensitivity is indicated by increasing numbers. This means that a high Ull Meter measure is anticipated as a high level of autonomic dysfunction (high sensitivity and low pain threshold) (Ballegaard et al., 2015). Home recording of PPS was conducted daily by the participants. A website was established for the study^1^, and each participant in the active group received a personal profile with login with the possibility to enter the PPS measures for personal track recording and furthermore to make ongoing professional surveillance possible and thus conduct proactive contact to the person in case of deviating or missing PPS measurements.

#### Baseline Characteristics

Body mass index was measured as weight in kilogram divided by height in squared meters; BP (mmHg) and pulse (beats/minute) were measured automatically in the supine position after 10 min of rest (Thuasne Ltd; automatic BP monitor); fasting lipids and kidney function were measured by routine methods.

#### Study Design

The study was a single-center, two-armed, parallel-group, observer-blinded, randomized (1:1), clinical superiority trial. The participants were enrolled at the research unit of the Department of Medicine, Endocrinology Unit, Herlev and Gentofte Hospital, Copenhagen, Denmark. The observation period was 6 months.

#### Minimizing Bias

The following precautions were made to minimize bias: (1) HbA1c was chosen as the primary outcome measure, i.e., a blood test result that is not affected by participant and/or researcher bias; (2) the PPS device was designed in a way making the measure non-visible before the end of each measurement for both instructor and participant; (3) the professional instructor measuring PPS was blinded to the result of the randomization; (4) the participants were instructed before randomization not to reveal the result of the randomization to the research personnel performing the follow-up investigation after the 6-month observation period.

#### Intervention Procedure

All participants received standard diabetes care according to national guidelines, which includes medical counseling by the study nurse, medical standardization by the study diabetologist, education in T2D, and lifestyle adjustments. At baseline, all participants were informed that they had an elevated PPS measure and that this reflected a physiological strain on the body, which they may not sense consciously but potentially was associated with the ANS function of the body, including the glucose metabolism, and a broad range of diabetes health risk factors, such as BP, HR, work of the heart, psychological well-being, serum lipids, BMI, inflammatory markers, and the level of physiological stress on the body.

#### Control Group

They received no further information or intervention, other than the information of the nature of the active treatment, i.e., a non-pharmacological self-care stress management program with the aim to reduce the PPS measure.

#### Active Group

All subjects were receiving a non-pharmacological self-care stress management intervention program (Ull Care^®^) and were instructed by a professional instructor (co-author SB). The program has been described previously in detail (Ballegaard et al., 2014; Bergmann et al., 2014) and has the following basic elements: (i) a self-care part; (ii) a professional instruction in the PPS measurement, cognitive reflection in relation to the PPS measure, and how to conduct sensory nerve stimulation; and (iii) continuous ongoing professional surveillance of the PPS measure, allowing the possibility for pro-active professional contact if PPS measurements are missing or deviating. The self-care part consists of two daily and mandatory efforts in the morning and evening: (i) perform PPS measurement, (ii) followed by sensory nerve stimulation as a mandatory stress-reducing procedure, and (iii) reflection on both the PPS level and general feeling of need for additional stress handling on a voluntary basis. We used the PPS measurement device (StressMeter^®^) developed to record the sensibility/activity of a polymodal nervous system at the most tender point on the sternum between costae 3 and 5, identified by finger pressure. First, the participant learns his or her pain threshold as the instructor applies a gradually increasing pressure during 5 s on the distal phalanges of the left index finger with the instrument, and the participant is instructed to say stop when the threshold of pain/discomfort is reached. If the instructor observes a withdrawal reflex (i.e., an involuntary muscle contraction of the muscles around the eyes, neck, or upper limb) before the participant says stop, the procedure is stopped as well. The procedure is then repeated at the most tender place on the sternum. The instrument displays a number on a scale from 30 to 100, where an increased sensibility is accompanied by an increasing PPS measure, meaning that a high PPS measure reflects a high sensitivity, or low threshold for pain or tenderness. The sensory nerve stimulation is conducted twice a day plus *ad hoc* in the case of an urgent need for stress reduction. Sensory nerve stimulation is done by applying non-painful pressure with a finger for 1 min on specific points on the body surface. A criterion for correct sensory nerve stimulation is when the person can observe that the tenderness of the point has been reduced after applying pressure for 20 to max of 60 s. If this result has not been achieved, the person is instructed to repeat the treatment. Locations used for the nerve stimulation include predefined tenders spot on the front and back parts of the chest wall and on arm and foot—which are all identified as tender by finger pressure. All subjects received a personal PPS measurement instrument, together with an instruction manual, and were initially instructed during a 2-h group session with 5–10 participants in each group. This included education in performing PPS measurement, sensory nerve stimulation, and the theoretical background for the measurement as well as the intervention. The participants were offered two individual appointments after 1 and 3 months and five phone contacts after 1, 3, 5, 6, and 10 weeks, and similarly a 2-h group session every other month. The participants were instructed to report their PPS measurements each day on their personal login on the website www.songdance.org. On the website, each participant was able to track results and changes in PPS during the intervention period. Further, the instructor was able to see these results and take action by contacting the participant, if the PPS measures did not change or deviated.

The repetitive sensory nerve stimulation has two goals: (1) to restore the nervous system’s ability to adapt through the repetitive stimulation of the diffuse noxious inhibitory control (DNIC) system (Ge et al., 2012) and (2) to reduce an elevated stress level through the repeated non-painful sensory stimulation of the polymodal nerve cell, causing a release of the oxytocin hormone (Uvnäs-Moberg et al., 2014). If possible, the spouse or cohabitant will be instructed to perform nerve stimulation on the person’s back, which may independently contribute to stress-relieving effects through a separate oxytocin release caused by the human care (Uvnäs-Moberg et al., 2014). If a spouse or cohabitant is not available, the person can perform the treatment himself with a ball (the person will receive instructions in the technique).

#### Algorithm for Evaluation of Glycated Hemoglobin After 3 Months of Observation

Both the control and active groups continued with the given medical treatment. If possible, the medication should remain stable during the first 6 months of observation. The participant and the person’s own doctor were informed that adjustment of diabetes medicine would be taken care of by the diabetologist assigned to the study.

#### Use of Glucose-Lowering Medication

1. The treatment target for glycemic control was 48 mmol/mol (6.5%).
2. Reduction in HbA1c to a level above or equal to 48 mmol/mol did not lead to change in medication.
3. In participants with an HbA1c below 48 mmol/mol, a further reduction of 3 mmol/mol required an evaluation with respect to reduction in anti-diabetes medication according to the Danish National Guidelines. This evaluation was performed by the study diabetologist (EE), who was blinded to the randomization.
4. If the participant experienced a hypoglycemic episode, she or he was asked to contact the study nurse, and the study diabetologist would consider whether change in glucose-lowering medication was needed.
5. If HbA1c increased more than 3 mmol/mol at any point at follow-up, the glucose-lowering medication was evaluated with respect to an increase in anti-diabetic medication and according to the judgment of the study diabetologist.

#### Statistics

Data were evaluated by parametric statistics using paired *t*-test, ANCOVA including baseline HbA1c results as dependent factor. Due to the nature of the study, i.e., a test of the conceptual association between a reduction of PPS (as a measure for improvement of hypothalamic autonomic dysregulation of glucose metabolism) and a reduction of HbA1c (as a measure for improved glucose metabolism), per-protocol (PP) analysis was used as the primary statistical analysis. However, intention-to-treat (ITT) analysis was performed in parallel using the basic observation carried forward (BOCF) method. It was expected that the 1st months of the study period were needed to render the subjects familiar with the intervention. Accordingly, a measurement of HbA1c was conducted after 3 months only with the aim to ensure proper diabetes medication, evaluated according to a predefined algorithm (see above). Only baseline and 6 months’ measurements were thus included in the effect measurements. However, as some participants had their diabetes medication changed during the study period, a supplementary statistical analysis was conducted excluding these participants to ensure that this did not cause a significant bias to the results. For correlation analyses, we used Pearson parametric correlation analysis. For group comparison of responder to non-responder ratio, we used the chi-square test; for group comparison of quality of sleep, we used Fisher’s exact probability test for three groups^2^. We used Cohen’s effect size to compare the active with control group using PP data. The effect size was evaluated according to Hedges and Olkin as the difference in mean change score from baseline to follow-up between the active and control groups and divided by the pooled standard deviation (Hedges and Olkin, 1985; Bech, 2007). In relation to clinically significant effects, the following has been proposed: effect size < 0.19 means minor clinically significant effect; 0.20–0.49, small effect; 0.50–0.79, medium effect; and ≥ 0.80, a large effect (Blais and Baer, 2010). Analyses were performed using the statistical package SPSS version 25. All statistical tests were two-sided, and *p*-values below 0.05 were considered statistically significant. All analyses were performed by an independent statistical expert blinded for the randomization.

#### Estimation of Sample Size

Calculation of sample size was based on the followed premises with respect to HbA1c: the minimal important difference was 4 mmol/mol; mean HbA1c as baseline was estimated to be 64 mmol/mol, and with standard deviation of 9 mmol/mol, alpha of 5%, and beta of 80%. This required 160 as the total sample size^3^.

### Pressure Pain Sensitivity as Measure of Autonomic Function

#### Design and Ethics

We completed a quality control assessment of two diagnostic methods used in the outpatient clinic assessment for chronic stress (PPS assessed by Ull Meter^®^) (Ballegaard et al., 2014) and ANSD (assessed by Vagus^®^) (Fleischer et al., 2015) at a tertiary hospital department of endocrinology and diabetes. The study was approved by the hospital quality control board (ID 18015437, Herlev and Gentofte Hospital, Copenhagen, Denmark). Accordingly, the study did not require submission to the local ethical committee for approval. As the study is part of the daily clinical routine work in the outpatient clinic, the patients have given informed consent as part of the routine work of the clinic. We recorded the use of lipophilic beta-adrenoceptor inhibitors that pass the blood–brain barrier, and we excluded patients treated with low lipophilic beta blockade (e.g., atenolol).

#### Study Population

Consecutive patients with T2D were recruited from the outpatient clinic during the last 3 months of 2017. There were no exclusion criteria.

#### Blinding

A study nurse conducted the PPS measurements and was blinded with respect to CAN score and medication as well as baseline status with respect to the T2D disease.

#### Procedure

Subjects rested for 10 min in the supine position before the PPS measurement and the subsequent CAN test. The procedures were supervised by an experienced research educated nurse.

#### Cardiovascular Autonomic Neuropathy Measurement

Cardiovascular autonomic neuropathy was evaluated using beat-to-beat variation with the Vagus^®^ test (Fleischer et al., 2015). It measures resting HR and computes beat-to-beat variation during three provocation tests: stand-up test, deep breathing for 1 min and with a frequency of six breaths/min, and forced expiration for 15 s against resistance of 40 mmHg, and 45 s of normal breathing (i.e., Valsalva maneuver), according to the Ewing criteria for ANSD testing (Bernardi et al., 2011). Each CAN test was evaluated as positive or negative with regard to autonomic dysfunction based on normal data for evaluation of a CAN score test provided by the manufacturer (Spallone et al., 2011). A total CAN score 0 means all tests are negative and indicates no CAN; CAN score 1 means one of the three tests is positive and is indicative of borderline CAN; CAN score 2 means a minimum of two of three tests are positive as indicative of definitive CAN.

#### Pressure Pain Sensitivity Measurement

The procedure of PPS measurement was identical to that described regarding study 1; see above.

#### Statistics

We used Pearson correlation coefficients to measure associations among quantitative outcome measures. Tests of significance used *t*-tests, *F*-tests from analysis of variance (ANOVA), chi-square test, and Fisher’s exact probability test. All statistics were calculated by SPSS version 25.

#### Sample Size

Sample size was set at a minimum of 20 patients in each of the three CAN categorization groups.

## Results

### Effect of Pressure Pain Sensitivity Reduction in Type 2 Diabetes

**FIGURE 2:**
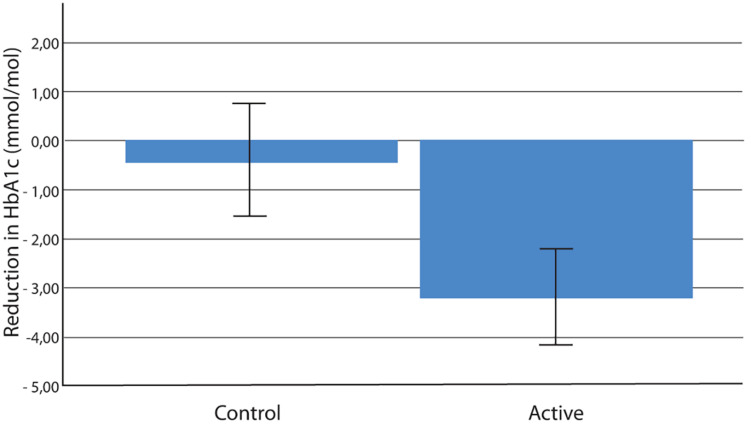
Reduction in HbA1c (mmol/mol) over 6 months in the active and control groups; mean ± SE; *p* = 0.035 (*n* = 52 active group, *n* = 60 control group). HbA1c, glycated hemoglobin.

Figure 1 shows the flow of the study. Thirty-six patients were excluded at baseline medical examination [PPS < 60 (*n* = 9), HbA1c > 75 mmol/mol (*n* = 2), HbA1c < 40 mmol/mol (*n* = 11), use of beta blockade medication (*n* = 6), type 1 diabetes (*n* = 1), and serious comorbidity (*n* = 7)]. The demographic characteristics of the included participants are listed in Table 1. PP analyses revealed that in the active group, mean HbA1c values changed from 53.8 to 50.5 mmol/mol (*p* = 0.001; *n* = 52), compared with the change of 53.8 to 53.4 mmol/mol in the control group (*p* = 0.7; *n* = 60; between group *p* = 0.036) (Figure 2 and Table 2). In the active group, the PPS measure declined from 76.6 to 56.1 arbitrary units (*p* < 0.001) and from 77.5 to 72.8 arbitrary units in the control group (*p* = 0.02; between group *p* < 0.001) (Table 2). The ITT analyses shown in Table 3 demonstrated similar results.

**TABLE 1:** Baseline characteristics of randomized participants.

	Active	Control
Number of persons	71	73
Age (years); mean (range)	64.3 (38–75)	65.6 (45–77)
Sex: male/female (number)	46/25	45/28
Diabetes duration (years); mean (range)	10.6 (1–25)	9.9 (1–24)
Primary diabetes control unit: general practitioner (%)	77%	78%
**Medication [number (%)]:**		
Metformin	54 (76)	56 (77)
Insulin	13 (18)	11 (15)
GLP-1 agonist	12 (17)	14 (19)
SGLT-2 inhibitor	8 (11)	11 (15)
DPP-4 inhibitor	10 (14)	20 (27)
Sulfonylurea	5 (7)	5 (7)
Statins	53 (75)	52 (71)
ACE/ARB	42 (59)	41 (56)
Diuretics	26 (37)	18 (25)
Calcium channel blockers	24 (34)	16 (22)
ASA	22 (31)	20 (27)
**Medical history [number (%)]:**		
Peripheral arterial disease	3 (4.2)	1 (1.4)
Previous myocardial infarction	4 (5.6)	2 (2.7)
Previous CABG or PCI	6 (8.5)	2 (2.8)
Previous stroke	2 (2.8)	1 (1.4)
Previous treated depression	7 (9.9)	9 (12)
Asthma	11 (15.5)	3 (4.2)
Previous cancer	8 (11.3)	5 (6.8)
Symptomatic neuropathy	10 (14)	14 (19)
**Pressure pain sensitivity (PPS) [arbitrary units; mean (SD)]:**		
Sternum	76.9 (13.3)	76.8 (13.6)
**Biochemistry [mean (SD)]:**		
Hemoglobin A1c (HbA1c; mmol/mol)	53.7 (8.6)	53.6 (10.5)
Creatinine (μmol/L)	75.7 (17.1)	77.6 (24.3)
Cholesterol (mmol/L)	4.09 (0.99)	4.09 (0.96)
LDL-cholesterol (mmol/L)	2.39 (3.52)	2.05 (0.77)
HDL-cholesterol (mmol/L)	1.22 (0.39)	1.28 (0.33)
Triglyceride (mmol/L)	2.16 (1.10)	1.83 (0.92)
**Physiology [mean (SD)]:**		
Systolic blood pressure (mmHg)	135 (15)	135 (17)
Diastolic blood pressure (mmHg)	79 (8)	79 (7)
Heart rate (beats/min)	70 (12)	68 (9)
BMI; [weight (kg)/height (m)^2^]	29.2 (4.7)	28.0 (4.5)

*No significant differences between the active and control groups. Medication: GLP, glucagon-like peptide; SGLT, sodium–glucose co-transporter; DDP, dipeptidyl peptidase; ACE, angiotensin-converting enzyme inhibitor; ASA, acetylsalicylic acid; ARB, angiotensin II receptor blocker. Medical history: CABG, coronary artery bypass grafting; PCI, percutaneous coronary intervention.*

**TABLE 2:** Effect of intervention (mean (SD)]: Per-protocol analysis.

	Baseline	6 months	*p*-value: baseline vs. 6 months	*p*-value: 6 months, active vs. control
*Primary outcome measure*				
HbA1c:				
Active	53.8 (8.4)	50.5 (7.0)	0.001	0.036
Control	53.8 (10.8)	53.4 (10.9)	n.s.	
*Secondary outcome measures*				
PPS (sternum):				
Active	76.6 (12.4)	56.1 (18.8)	< 0.001	< 0.001
Control	77.5 (13.6)	72.8 (17.5)	0.019	
Weight:				
Active	86.2 (19.0)	87.1 (14.9)	n.s.	n.s.
Control	87.0 (15.9)	87.9 (16.4)	n.s.	n.s.
BMI:				
Active	29.5 (4.2)	29.4 (4.3)	n.s.	n.s.
Control	28.4 (4.4)	28.7 (4.8)	n.s.	n.s.

*n = 52 active; n = 60 control. n.s., not significant; HbA1c, glycated hemoglobin A1c; PPS, pressure pain sensitivity; BMI, body mass index.*

**TABLE 3:** Effect of intervention [mean (SD)]: Intention-to-treat analysis.

	Baseline	6 months	*p*-value: baseline vs. 6 months	*p*-value: 6 months, active vs. control
*Primary outcome measure*				
HbA1c: Active Control	53.3 (8.4) 53.6 (10.6)	50.9 (7.4) 53.2 (10.7)	< 0.001 n.s.	0.049
*Secondary outcome measures*				
PPS (sternum): Active Control	76.9 (13.3) 76.8 (13.6)	61.9 (20.4) 73.0 (16.7)	< 0.001 0.021	< 0.001
Weight: Active Control	90.7 (16.5) 86.0 (15.7)	90.3 (16.6) 86.7 (16.2)	n.s. n.s.	n.s. n.s.
BMI: Active Control	29.7 (4.5) 28.4 (4.4)	29.6 (4.5) 28.7 (4.7)	n.s. n.s.	n.s. n.s.

*n = 71 active; n = 73 control. n.s., not significant; HbA1c, glycated hemoglobin A1c; PPS, pressure pain sensitivity; BMI, body mass index.*

**FIGURE 3:**
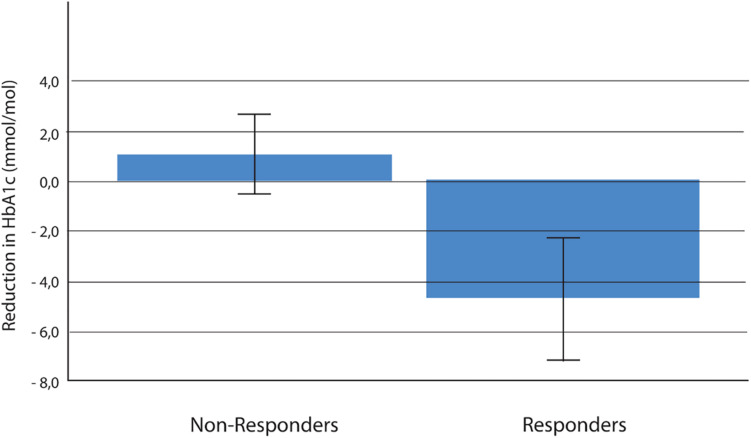
Reduction in HbA1c (mmol/mol) over 6 months among responders (a decrease in PPS ≥ 15 arbitrary units) versus non-responders (a decrease in PPS less than 15 arbitrary units); (active and control group together) mean ± SE; *p* < 0.001 (*n* = 52 in responder group and *n* = 60 in non-responder group). HbA1c, glycated hemoglobin; PPS, pressure pain sensitivity.

We evaluated the effect of reduced PPS in the total group of participants (i.e., treatment and control groups together), which was possible because of the non-pharmacological nature of the intervention. Responders were participants who obtained the predefined minimum difference of changes to PPS during the intervention period, i.e., 15 arbitrary units or more (non-responders having a PPS reduction to less than 15). Thirty-five of 52 participants in the active group (67%) were responders, compared with 17 of 60 in the control group (28%; between group *p* = 0.0001). Odds ratio for being a responder in the active group compared with the control group was 5.20 (95% confidence interval: 2.3–11.6). During the intervention period, the responder group obtained a significant reduction in HbA1c (mean -0.49 mmol/mol, SD 9.1; *n* = 52; *p* < 0.001), whereas the non-responder group was unchanged (mean + 1.0 mmol/mol; SD 6.0; *n* = 60; between group *p* < 0.001) (Figure 3).

**FIGURE 4:**
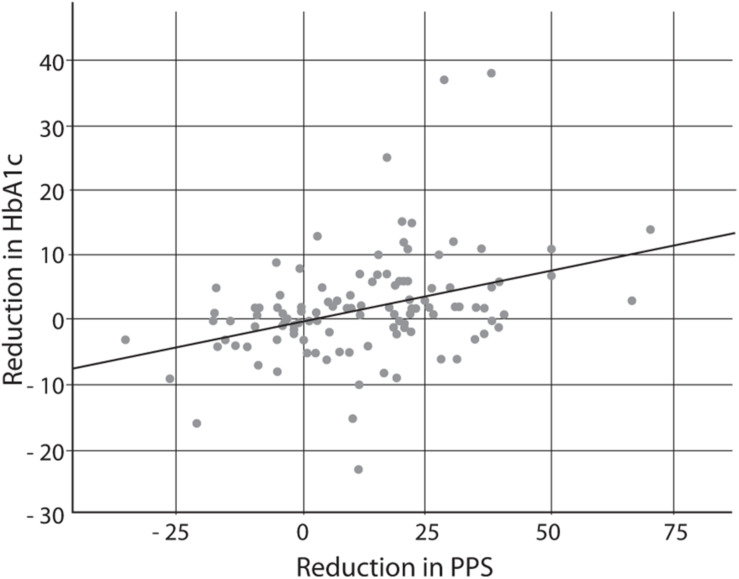
Correlation between reduction in PPS (arbitrary units) and reduction in HbA1c (mmol/mol) over the 6-month study period, including both the active and control groups; *n* = 112; *r* = 0.37; *p* < 0.001. PPS, pressure pain sensitivity; HbA1c, glycated hemoglobin A1c.

According to the protocol, we regarded a reduction of 4 mmol/mol as the minimal clinically relevant reduction of HbA1c. When the clinical effect was evaluated as Cohen’s effect size, the effect size was 0.37 when the active group was compared with the control group and 0.74 when the group of responders was compared with the group of non-responders. Including all participants, there was a significant and positive correlation between changes of PPS and changes of HbA1c during the intervention period (correlation coefficient *r* = 0.37; *p* < 0.001, *n* = 112) (Figure 4).

No adverse effects were observed. However, two participants discontinued the treatment for reasons of inconvenience. With respect to compliance regarding home PPS measurement in the active group, the mean number of days with at least one PPS home measurement was 164 out of in total 182 observation days [i.e., compliance rate: mean (SD) 90% (14.7%); range 44–100%] (*n* = 52).

We completed a sub-analysis of participants who did not change their diabetes medication during the 6-month experimental period. In the active group, two individuals raised and two individuals lowered their medication, as compared with three in the control group who raised their medication and one reducing. This rendered 48 participants in the active group and 52 participants in the control group. The sub-analysis did not change the results: mean HbA1c declined 3.1 mmol/mol (SD 7.0) in the active group (*n* = 48), compared with an increase in the control group (*n* = 56) of + 0.2 mmol/mol (SD 7.6; between group *p* = 0.025). Furthermore, in this subgroup of participants, the correlation between reduction of PPS and reduction of HbA1c during intervention did not change (correlation coefficient *r* = 0.37; *p* < 0.001).

Since the PPS measure partly depends on a sensation, the existence of diabetic neuropathy could in theory influence this measure. In the active/control groups, 14/19% reported clinically sensory neuropathy at baseline (*p* > 0.1). Analyzing the total group of people included in the study demonstrated no significant difference in PPS at baseline nor after 6 months’ follow-up whether neuropathy or not (between group *p* > 0.1).

The use of GLP-1 receptor agonists (GLP-1 RAs) has been found to potentially affect the ANS (Hansen et al., 2019). Eighteen percent of the population received GLP-1 RA. A sub-analysis comparing baseline PPS in those treated with GLP-1 RA versus those who did not did not show any significant difference and a sub-analysis excluding GLP-1 RA-treated people did not change the main results.

### Pressure Pain Sensitivity as Measure of Autonomic Function

The demographic characteristics of the study population of people with T2D are presented in Table 4 and were all evenly distributed in the three groups based on CAN status with the exception of BMI. Fifty-five percent of patients had elevated PPS (≥ 60 arbitrary units).

**TABLE 4:** Baseline demographic characteristics of study groups (study 2).

	All	Patients with no positive CAN test	Patients with one positive CAN test	Patients with 2 or 3 positive CAN tests	*p*
n	76	20	32	24	
Age (years), mean (SD)	60 (9.7)	63 (10.8)	60 (7.9)	60 (11.1)	0.46
Female/male,%	47/53	45/55	51/49	42/58	0.58
BMI (kg/m^2^), mean (SD)	31 (5.1)	31 (3.8)	30 (4.9)	34 (5.8)	0.012
HbA1c, mmol/mol, mean (SD)	62 (13.8)	64 (16.9)	63 (12.6)	59 (12.4)	0.36
Duration of type 2 diabetes (years), mean (SD)	13 (5.4)	12 (5.3)	14 (5.7)	12 (5.2)	0.46
Withdrawal reflex; n (%)	68 (88%)	18 (90%)	30 (91%)	20 (83%)	0.68
PPS finger, arbitrary units, mean (SD)	32 (4.5)	30 (1.4)	32 (5.9)	31 (3.9)	0.26
**Comorbidities**, n (%)					
CHD, n (%)	20 (26%)	5 (25%)	12 (36%)	3 (13%)	0.17
Cerebrovascular disease, n (%)	11 (14%)	2 (8%)	6 (14%)	3 (11%)	0.65
PAD, n (%)	2 (3%)	0 (0%)	1 (3%)	1 (4%)	0.67
Nephropathy, n (%)	30 (39%)	7 (35%)	11 (33%)	12 (50%)	0.34
Retinopathy, n (%)	20 (26%)	5 (25%)	8 (24%)	7 (29%)	0.93
Neuropathy, n (%)	20 (26%)	5 (25%)	8 (24%)	7 (29%)	0.93
Hypertension, n (%)	70 (91%)	18 (90%)	32 (100%)	20 (83%)	0.22
**Anti-diabetic medication**					
Metformin, n (%)	60 (78%)	16 (80%)	24 (73%)	20 (83%)	0.57
SGLT-2 antagonist’ n (%)	25 (33%)	6 (30%)	10 (30%)	9 (38%)	0.75
GLP-1 analog, n (%)	51 (66%)	12 (60%)	19 (58%)	20 (83%)	0.12
Supplementary insulin therapy, n (%)	47 (61%)	13 (65%)	19 (58%)	15 (63%)	0.80
**Other medications**, n (%)					
Antihypertensive medication, n (%)	68 (89%)	18 (90%)	32 (100%)	20 (83%)	0.22
ACE/ARB inhibitor, n (%)	60 (78%)	13 (65%)	29 (88%)	18 (75%)	0.15
Beta-blockers, n (%)	26 (34%)	6 (30%)	12 (36%)	8 (33%)	0.85
Lipid-lowering drugs, n (%)	66 (86%)	17 (85%)	29 (88%)	20 (83%)	0.91

*Table includes all patients who concluded all three CAN tests only (n = 76). No significant differences with respect to the included demographic variables were found between the groups of patients with CAN score 0, 1, or 2. SD, standard deviation; PPS, pressure pain sensitivity; CAN, cardiovascular autonomic neuropathy; CHD, cardiovascular heart disease; PAD, peripheral arterial disease; SGLT, sodium–glucose co-transporter; GLP, glucagon-like peptide; ACE, angiotensin-converting enzyme inhibitor; ARB, angiotensin II receptor blocker.*

#### Pressure Pain Sensitivity and Heart Rate

The resting PPS of the chest bone correlated with resting HR in the absence of beta-adrenoceptor blockade (*r* = 0.35, *p* = 0.006, *n* = 61), but not in the presence (*r* = 0.02, *p* = 0.92; *n* = 32). Thus, mean HR was 71 and 79 beats/min in the presence and absence, of beta-adrenoceptor blockade, respectively (*p* = 0.0245), while mean PPS was identical in the two groups (63 arbitrary units in both; *p* = 0.9710).

#### Pressure Pain Sensitivity and Cardiovascular Autonomic Neuropathy

Seventy-six participants completed all three CAN tests, with CAN score 0 in 20 participants (26%; no autonomic dysfunction), 1 in 32 participants (42%; borderline autonomic dysfunction), and 2 or 3 in 24 participants (32%; definite autonomic dysfunction). PPS increased with CAN score (mean PPS values 54, 60, and 71 in the three groups with CAN score 0, 1, and 2 or 3, respectively (*p* = 0.007, *n* = 76) (Table 5). Looking at the individual CAN tests and recording a positive or negative CAN test, the mean PPS (± SD) were as follows: standing up: CAN score negative/positive: PPS = 54.1 (± 18.0; *n* = 37)/68.8 (± 14.9; *n* = 39), respectively (*p* < 0.0001); breathing: CAN score negative/positive: PPS = 62.4 (± 18.7; *n* = 49)/61.7 (± 17.6; *n* = 27), respectively (*p* = 0.88); Valsalva: CAN score negative/positive: PPS = 59.8 (± 16.5; *n* = 55)/68.5 (± 21.3; *n* = 21), respectively (*p* = 0.10). Table 6 shows the PPS values for all participants who concluded each individual CAN score test. The associations between PPS and the CAN score outcomes were not significantly influenced by beta-receptor blockade (all *p* > 0.1). Twenty-six percent of the patients reported sensory neuropathy. PPS was not significantly different compared with that in patients with and without neuropathy (*p* > 0.1).

**TABLE 5:** Pressure pain sensitivity for all patients who concluded all three CAN tests.

	Patients with no positive CAN test (*n* = 20)	Patients with one positive CAN test (*n* = 32)	Patients with two or three positive CAN tests (*n* = 24)	All patients (*n* = 76)	*p*-value
PPS, arbitrary units Mean (SD)	54 (14.4)	60 (19.9)	71 (15.9)	62 (18.4)	0.007
Resting heart rate (beats/min) Mean (SD)	72 (15.3)	71 (9.7)	87 (15.8)	76 (15.1)	0.000
Resting heart rate > 100 beats/min n (%)	0 (0%)	0 (0%)	6 (25%)	6 (8%)	0.000

*For the three categories of patients who had (i) no, (ii) one, or (iii) more than one positive CAN test and for all patients. PPS, pressure pain sensitivity; CAN, cardiovascular autonomic neuropathy.*

**TABLE 6:** Pressure pain sensitivity for the positive/negative outcome of each of the three CAN score tests.

Vagus test completed	Vagus Test tested	CAN Score	PPS; mean (SD)	p-value	Number
Standing-up test (tilt) (*n* = 95)	1	Negative	56 (20.3)	0.000	49
		Positive	70 (15.3)		46
Breathing test (*n* = 93)	2	Negative	64(19.8)	0.64	55
		Positive	62(19.7)		38
Valsalva test (*n* = 78)	3	Negative	60(16.5)	0.067	57
		Positive	69(21.3)		21

*For all the patients who concluded that specific CAN score test but not necessarily all three tests. PPS, pressure pain sensitivity; CAN, cardiovascular autonomic neuropathy.*

#### Compliance of Pressure Pain Sensitivity and Cardiovascular Autonomic Neuropathy Measurements

All 111 participants concluded the PPS measurement, and 76 patients (68%) concluded all three CAN tests from the Vagus^®^ test procedure (between group *p* < 0.0001). Ninety-five participants concluded the stand-up test and 93 the breathing test. The Valsalva maneuver that technically was difficult and resulted in dropouts was carried out only in 78 participants.

#### Adverse Events

General discomfort during the performance of the Valsalva test led 33 out of 111 participants (30%) to discontinue this test. No adverse effects were observed from the PPS measurements.

## Discussion

### Main Findings

The present investigation addresses a new approach to the treatment of T2D that focuses on the cerebral regulation of glucose homeostasis.

First, we conducted the RCT as a single-center, two-armed, parallel-group, observer-blinded, randomized (1:1), clinical superiority trial. It did not reject the hypotheses that (1) the PPS-guided non-pharmacological intervention lowers HbA1c and PPS in people with T2D and with an elevated PPS (i.e., PPS > 60 arbitrary units), and (2) a reduction of an elevated PPS was closely associated with a reduction in HbA1c. For the participants who obtained a predefined minimum improvement in PPS of 15 arbitrary units regarded as clinically relevant during the intervention period and thus were regarded as responders, HbA1c levels decreased by approximately 6 mmol/mol (11%), compared with the group of non-responders.

Second, we focused on the association between PPS and ANSD and the possible influence by beta-receptor blockade, to elucidate the consequences of reducing PPS and HbA1c and the association between the two in T2D. We demonstrated (1) that elevated PPS is associated with elevated HR and autonomic sympathetic predominance measured by HRV in T2D and (2) that PPS is not influenced by beta-adrenergic receptor activity, as neither the afferent nor efferent loop of the PPS regulation was affected by beta-receptor blockade medication. HR was influenced by the beta blockade medication, which typically reflects the efferent sympathetic stimulus from the brain. Furthermore, the association between PPS and HR was present in non-beta-receptor blockade medication users only. The findings support the previous assessment of intervention in patients with chronic ischemic heart disease that PPS may be considered a measure of ANS function.

Thus, the present findings are consistent with the hypotheses (1) that the PPS-guided non-pharmacological intervention lowers HbA1c and PPS and (2) that a reduction of elevated PPS, as measure of ANSD, reflects long-term lowering of glucose levels, measured by HbA1c as evidence of improved glucose homeostasis and of a close association between reduction of PPS and reduction of HbA1c, the latter a measure of improved cerebral glucose metabolic control.

As the inclusion criterion of participation was ANSD at baseline, defined as an elevated PPS measure of at least 60 units, these findings underscore the association between ANSD as a brain activity measured by PPS and the control of T2D.

### Cerebral Regulation of Blood Glucose

It has been suggested that persistent elevation of sympathetic activity is a key to the pathogenesis of T2D (Güemes and Georgiou, 2018; Lundqvist et al., 2019) and that peripheral nerve stimulation aimed at down-regulating sympathetic activity may be a future therapeutic approach to T2D (Güemes and Georgiou, 2018). As such, the present investigation supports the concept of an association between cerebral ANSD, as measured by PPS, and glucose regulation in patients with T2D, as well as the utility of non-pharmacological intervention, using peripheral sensory nerve stimulation to reduce excess cerebral sympathetic activity and restore normal cerebral autonomic glucose regulation.

### Pressure Pain Sensitivity as Measure of Cerebral Autonomic Function

**FIGURE 5:**
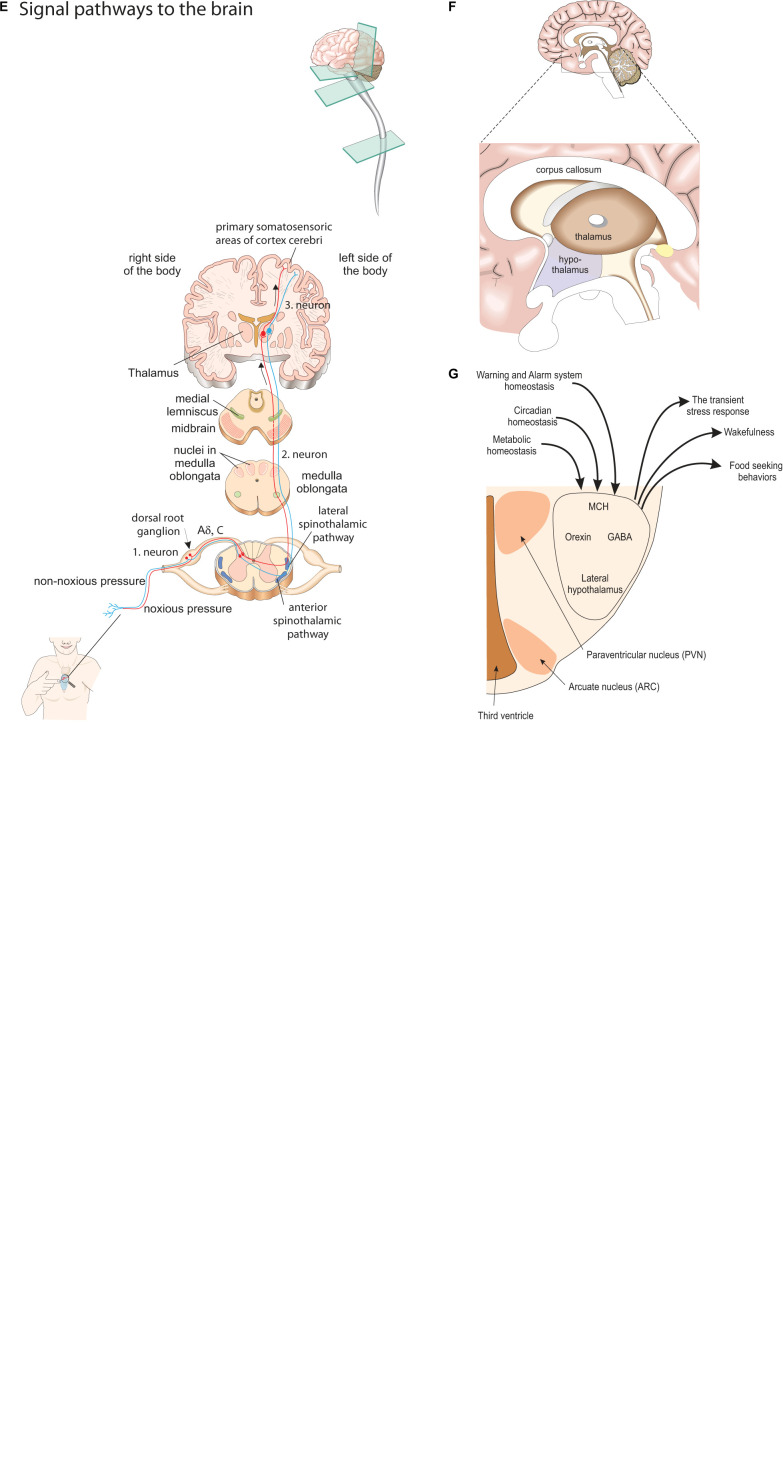
Overview of the neurophysiological pathways of the PPS concept. **(A)** Measurement site: pressure pain sensitivity is measured at the periosteum of the chest bone, rather than that of the skin. **(B)** Distinction between the polymodal receptor (periosteum) and the mechano receptor (skin): the dual function of the polymodal sensor cell in contrast to the other local sensory cells. Pressure pain threshold changes by the level of sympathetic activity. **(C)** Reactions of the polymodal sensor cell: the reactions of the polymodal sensor cell in response to the degree of applied pressure. **(D)** Two kinds for signals from periosteum to the brain: noxious (red, lateral spinothalamic tract) and non-noxious signal (blue, anterior spinothalamic tract) from A-delta and C-fibers use different pathways to the brain. **(E)** Signal pathways to the brain: transmission pathways of noxious and non-noxious sensory nerve stimulation to thalamus and somatosensoric areas of the cerebral cortex. **(F)** Sagittal view of brain with thalamus and hypothalamus: thalamus and hypothalamus. Both kinds of signals from the A-delta and C-fibers reach the thalamus and from there transmit to the lateral hypothalamus. **(G)** Frontal view of lateral hypothalamus: the lateral hypothalamus with the close association between homeostatic regulation of metabolism, wakefulness and Warning and Alarm system by the Orexin system, GABA (gamma aminobutyric acid) and MCH (melanin concentrating hormone).

The association between PPS and the autonomic warning and defense system is the key to the understanding of the present concept of changes in PPS reflecting changes in sensitivity of the autonomic warning and defense system regulation by the regulatory center of the stress response, i.e., the hypothalamus. Pressure pain perception at the periosteal bone depends on activation of nociceptive neurons, i.e., terminals of myelinated A-delta and unmyelinated C-fiber neurons (Nencini and Ivanusic, 2016; Figures 5A–G), and perhaps also of glial cells of the dermis (Abdo et al., 2019; Doan and Monk, 2019). The neurons express a wide range of receptors and ion channels sensitive to noxious stimuli. The neurons transform stimuli into electrical signals directed toward the central nervous system. The most important group of ion channels is members of the transient receptor potential (TRP) family. The channels are responsible for the raised mechano-sensory and temperature thresholds that ensure reflexive withdrawal from danger during transient stress, e.g., as signaled by pain (Figures 5A–G) (i.e., the withdrawal reflex).

While this reflex is an essential part of warning and defense mechanisms (Veldhuis et al., 2015), the sensitivity of the TRP ion channels is modulated by the sympathetic nervous system at multiple stages of reflex control, mediated anatomically as a loop, with an afferent signal, a central part, and an efferent signal (Veldhuis et al., 2015). The lateral hypothalamus has been found to be integrated in the center of modulation of this reflex (Franco and Prado, 1996). Previously, it was found that the PPS measure is closely associated with this reflex, as observed in transient as well as persistent stress (Ballegaard et al., 2009), and as confirmed here by the association of the PPS measure with the noxious withdrawal reflex (e.g., eye blink reflex) in 88% of the participants. For the patients in whom the noxious withdrawal reflex was not used as the termination point for the application of gradual increased pressure by the measurement device, the person has verbally said “stop” due to sensation of discomfort/pain in response the pressure. As such, in this situation, the PPS measure will always be higher than the threshold for the noxious withdrawal reflex.

Noxious withdrawal reflex hypersensitivity and thus pressure pain hypersensitivity are maintained by TRP channel sensitization, as seen in disease conditions related to persistent stress and autonomic dysfunction, including allodynia (i.e., perception of a non-painful stimulus as painful) in diabetes (Dias et al., 2019). Sensory hypersensitivity has also been reported as early in life as in preschool children as a sign of persistent stress and a precursor of anxiety disorders (Carpenter et al., 2019).

The down-regulation of pressure pain hypersensitivity and thus the noxious withdrawal reflex is mediated by the same autonomic reflex loop (Ge et al., 2012; Suokas et al., 2012). The afferent leg of the pain loop has been found to be modulated by non-pharmacological electrical sensory nerve stimulation both in patients with painful diabetic neuropathy (Tesfaye et al., 2010) and in people with chronic angina pectoris (Richner et al., 2019). Non-painful cutaneous sensory nerve stimulation has been found to release the hypothalamic peptide oxytocin that reduces pain sensation, reduces stress, and restores normal autonomic function (Uvnäs-Moberg et al., 2014).

In order to localize the site of PPS regulation, we used beta-receptor blockade medication, as generally held to inhibit the efferent stress response loop mediated by the autonomic sympathetic nervous system. Two previous studies revealed unaffected PPS by this medication, while the secondary effects from a reduction of an elevated PPS on depression score and BP response to tilt table testing were inhibited (Ballegaard et al., 2016). Beta-adrenoceptors are widely distributed in the periosteum of bone (Khosla et al., 2018) and in the brain, including the hypothalamus, hippocampus, and prefrontal cortex (Rainbow et al., 1984), but are not engaged in the activity of the orexin cells of the lateral hypothalamus (Murakami et al., 2015). In this area of the brain, the orexin cells regulate stress responsiveness, metabolic and circadian homeostasis, and pain perception (Arrigoni et al., 2019) but remain unaffected by beta blockade (Murakami et al., 2015). We interpret the data to indicate that beta-adrenoceptors do not engage the afferent and efferent pathways connecting the source of the chest bone PPS to cerebral regulatory centers of PPS, while they do engage the association between PPS and HR.

Altogether, the observations are compatible with the hypothesis that the regulation of autonomic warning and defense systems, and hence of PPS, is integrated within the orexin system of orexin-A/hypocretin-1 and orexin-B/hypocretin-2 cells anatomically placed in the lateral part of the hypothalamus (Murakami et al., 2015) and with the claim that intervention by peripheral sensory nerve stimulation engages the afferent arm of this reflex loop. The result, PPS, is a measure of the pain threshold that reflects the efferent impulse. However, this conclusion is clearly based on indirect evidence, where direct measures of the hypothalamus by *in vivo* brain imaging studies are warranted. An association between the PPS measure and glucose metabolic homeostasis in the present study is not surprising, with PPS as indicator of autonomic warning and defense system activity, and HbA1c as indicator of autonomic glucose homeostasis.

### Pressure Pain Sensitivity Reduction for Improvement of Glucose Homeostasis

It is well known that ANSD is associated with obesity and sleep apnea (Peterson et al., 1988; Kakutani-Hatayama et al., 2020), both closely linked to the development of T2D. The common denominator probably is the link between ANSD and insulin resistance as shown by Saito et al. (2015). The reduction of HbA1c of in the present study might be taken as reduced insulin resistance, as the effect on HbA1c was seen also in participants with unchanged diabetes medication. Unfortunately, we have no direct measures of insulin resistance or insulin levels. However, in a previous study of healthy office workers during intervention, a trend was found toward an association between a reduction of elevated PPS and reduction of insulin resistance, measured as Homeostatic Model Assessment of Insulin Resistance (HOMA-IR) (Ballegaard et al., 2014).

Autonomic nervous system dysfunction and elevated HbA1c are independent risk factors for development of ischemic heart disease and premature death in diabetes patients. In previous RCT studies using the present intervention to reduce elevated PPS, beneficial effects were found on a broad range of clinically relevant diabetes and cardiovascular risk factors (Ballegaard et al., 2014; Bergmann et al., 2014). In an RCT, we recently demonstrated that long-term follow-up revealed reduced overall mortality after successful intervention with reduced PPS in patients with ischemic heart disease, both when comparing the active treatment and control groups and when we compared the treatment group with the general Danish population (unpublished).

Weight changes due to changes life style or diet during the study potentially could have influenced the ANS (Peterson et al., 1988). Thus, in a previous study on healthy office workers using the same intervention, we did find a significant association between reduction of an elevated PPS and reduction of BMI and visceral fat (Ballegaard et al., 2014). However, we found no changes of body weight in the present study (Table 2, 3), which means that reductions of neither HbA1c nor PPS can be explained by weight reduction.

As such, the findings of the present study support the concept that the central ANS actively is engaged in the regulation of glucose metabolism and that the ANSD is important to the pathophysiology of T2D. It also shows that PPS-guided non-pharmacological self-care-based intervention lowers ANSD and thus improves glucose homeostasis.

### Effect Size and Compliance

The effect of the present intervention, during 6 months of intervention, on the value of HbA1c is moderate (i.e., 4–6 mmol/mol), compared with the effect of classical pharmaceutical treatment, for example, the 12 mmol/mol change seen after treatment with metformin (Hirst et al., 2012). It is noteworthy that the treatment is without risks of side effects. Furthermore, the Cohen effect size (Hedges and Olkin, 1985; Bech, 2007) was 0.37 when we compared the treatment group with the control group, and 0.74 when we compared the group of responders with the group of non-responders, indicating small and moderate effects, respectively (Blais and Baer, 2010). We note that to obtain approval for a new anti-depressive medicine, the US Federal Drug Administration requests a Cohen effect size of at least 0.3. This implies that the present novel approach to the regulation of glucose homeostasis and treatment of T2D is clinically relevant. Moreover, due to the nature of the intervention, it cannot be ruled out that longer periods of intervention may lead to further reductions of HbA1c.

With respect to compliance, we recorded at least one daily home PPS measurement in 90% of the participants during the 6-month study period. With respect to the use of the home measure, a high compliance is required to ensure a solid basis of (1) daily biofeedback-guided stress handling, with the evidence from previous clinical experience suggesting a minimum of three measurements per week (corresponding to a compliance rate of approximately 45%), and (2) the ongoing professional surveillance, where a minimum of one measurement per week seems needed (corresponding to an approximate compliance rate of 15%). Thus, the present treatment group had high compliance to the requirements of PPS home measurement.

### Strengths and Limitations

#### Strengths

(1) The primary outcome measure chosen (HbA1c) is not influenced by participant or researcher bias; (2) the hypothesis of PPS as a measure of ANSD was established prior to the study in people with ischemic heart disease and was replicated in people with T2D in the present study, as was the hypothesis that a reduction of an elevated PPS is associated with reduction of HbA1c.

#### Limitations

(1) The study was designed as a single, blinded, randomized trial that may introduce a bias. Due to the nature of the study, participants could not be blinded regarding PPS home measurements and the administered intervention. However, the study nurse who performed all outcome measurements at the visits to the research unit paid special attention to the blinding to randomization. Although it has a subjective component, the measurement of PPS is connected to the observation of a noxious withdrawal reflex (e.g., the startle reflex), which is an objective observation and was observed in 90% of the participants; (2) the dropout rate was more than 25% in the treatment group, which seemed higher than in the control group, but the difference was insignificant and the main reason for dropout was development of severe disease during the study period (e.g., diagnosed cancer, prolonged infection, and operation for intermittent claudication). Only two persons dropped out because of non-compliance to the treatment; (3) the control group received the information that their condition potentially was associated with a negative impact on their disease and accordingly did not represent a fully inactive control group. However, the statistical findings are robust and with highly significant between-group differences. Furthermore, there are findings that the responder group (i.e., participants who obtained a predefined reduction of PPS ≥ 15 arbitrary units) had a pronounced effect in terms of reduced HbA1c independently of being randomized to the treatment or control group.

In terms of likelihood of becoming a responder, the likelihood was five times higher for the active group, compared with the control group. The difference is consistent with the conceptual understanding that the reduction of HbA1c primarily is associated with a reduction of PPS and thus with ANSD. (4) The intervention is a “package” including (i) repeated biofeedback PPS measurement and cognitive reflection, (ii) nerve stimulation, and (iii) professional surveillance. The intervention can also be regarded as containing this specific content, but also including a non-specific part as indicated by the observation that 28% of the persons in the control group achieved a reduction in PPS ≥ 15 arbitrary units and were thus identified as a responder. We are aware that we were unable to distinguish between the relative impacts of each of the three elements in the specific part. However, the contribution of the specific part probably is high, since the chance of being a responder was five times higher in the treatment group. In agreement with this, the “package” has been used in several RCTs, and each time, a substantial and significant reduction of elevated PPS was observed (Ballegaard et al., 2014; Bergmann et al., 2014).

The non-specific part included increased focus and personal responsibility regarding the diabetes as such when randomized to the treatment group. All participants were informed at baseline that the increased PPS potentially is associated with elevated long-term glucose regulation and poorer prognosis. This information may affect non-specific personal efforts, with the aim of reducing blood glucose. The control group demonstrated a reduction of PPS, with close to 30% being responders. In this context, it has been shown that focus on continuous glucose home measurement and a sentiment of increased empowerment may improve glucose metabolism (Wada et al., 2020), as well as continued professional surveillance using telemedicine also seems to improve glucose metabolism (Faruque et al., 2017). This indicates the potential power of the non-specific part. However, in summary, it is likely that all three specific parts of the intervention contribute to a major effect as compared with non-specific elements, as indicated by the five times increased chance of being a responder among patients in the active group, when compared with the patients of the control group.

We consider it a strength that we tested only lipophilic beta-adrenoceptor inhibitors that pass the blood–brain barrier. However, the resting HR of the patients receiving beta-receptor blockade treatment was relatively high, which may indicate only partial beta-receptor blockade. This may explain the lack of influence from beta-receptor blockade on the association between PPS and CAN score. It may also be explained by the observation that T2D patients in general have relatively high resting HRs, probably due to some degree of autonomic dysfunction in the majority of the patients.

### Clinical Perspectives

Central autonomic dysfunction is key to the understanding of glucose metabolic pathology and thus to T2D that is not addressed by present pharmaceutical means. The PPS tool and the associated treatment are applicable to the assessment of central autonomic function in patients with T2D, for therapeutic and preventive purposes, with a high patient compliance and no risk of side effects.

Autonomic dysfunction is also an important aspect in many everyday human complaints, common diseases, and even life-threatening health conditions, at present only treated by pharmaceutical or surgical means, which do not address the autonomic dysfunction aspect of the condition. The PPS tool and the associated intervention may be applicable to these situations as well.

## Conclusion

Here, we present a novel, non-pharmacological treatment of T2D, aimed at the reduction of cerebral ANSD as a means to improved glucose metabolism. Patients with T2D with elevated PPS, as an indication of cerebral ANSD, benefit from intervention based on a combination of repeated home measurements of PPS for track recording and cognitive reflection in terms of sufficiency of present own efforts, peripheral nerve stimulation for reducing the PPS, and professional surveillance with the possibility to act proactively if measures were missing or deviating. The participants integrated these procedures into their daily life, with substantial reduction of PPS and a clinically relevant long-term reduction of glucose concentration.

## Data Availability Statement

The raw data supporting the conclusions of this article will be made available by the authors, without undue reservation.

## Ethics Statement

The studies involving human participants were reviewed and approved by the Ethical Committee on Health Research Ethics, Region of Copenhagen, Denmark (ID) (identifier: H 17034836). The patients/participants provided their written informed consent to participate in this study.

## Author Contributions

JF has been a major contributor of all aspects of the manuscript. EE has been the blinded diabetes specialist of the study. AG had a major role in the interpretation of the results and preparation of the manuscript. SB is the inventor of the device and internvetion used, and had a major role in design of the study, education of the patients in the active group, and discussion of the manuscript. FG, CP, SKH, EE, and TW had an role in interpretation of the results and preparation of the manuscript. BK, CS, and SKs contribution are statistical analysis. All authors contributed to the article and approved the submitted version.

## Conflict of Interest

SB is a shareholder in the company UllCare A/S that holds the patent for the PPS-measurement device. He did not participate in patient selection, examinations, or evaluation of the examinations. The remaining authors declare that the research was conducted in the absence of any commercial or financial relationships that could be construed as a potential conflict of interest.

## Footnotes

1 www.songdance.org

2 http://vassarstats.net/

3 https://biostat.app.vumc.org/wiki/Main/PowerSampleSize (version 3, 2014)

## Abbreviations

ACE: angiotensin-converting enzyme inhibitor
ANS: autonomic nervous system
ANSD: autonomic nervous system dysfunction
ARB: angiotensin II receptor inhibitor
ASA: acetylsalicylic acid
BOCF: basic observation carried forward
BMI: body mass index
CABG: coronary artery bypass grafting
CAN: cardiovascular autonomic neuropathy
COPD: chronic obstructive pulmonary disease
CNS: central nervous system
DDP: dipeptidyl peptidase
DNIC: diffuse noxious inhibitory control
GLP: glucagon-like peptide
HbA1c: glycated hemoglobin
ITT: intention to treat
PCI: percutaneous coronary intervention
PP: per protocol
PPS: pressure pain sensitivity
RCT: randomized controlled trial
T2D: type 2 diabetes
TRP: transient receptor potential.
